# Therapies for Chronic Allograft Rejection

**DOI:** 10.3389/fphar.2021.651222

**Published:** 2021-04-15

**Authors:** Min Young Kim, Daniel C. Brennan

**Affiliations:** Department of Internal Medicine, Johns Hopkins School of Medicine, Baltimore, MD, United States

**Keywords:** transplantation immunology, kidney transplantation, antibody formation, graft rejection, antirejection therapy

## Abstract

Remarkable advances have been made in the pathophysiology, diagnosis, and treatment of antibody-mediated rejection (ABMR) over the past decades, leading to improved graft outcomes. However, long-term failure is still high and effective treatment for chronic ABMR, an important cause of graft failure, has not yet been identified. Chronic ABMR has a relatively different phenotype from active ABMR and is a slowly progressive disease in which graft injury is mainly caused by *de novo* donor specific antibodies (DSA). Since most trials of current immunosuppressive therapies for rejection have focused on active ABMR, treatment strategies based on those data might be less effective in chronic ABMR. A better understanding of chronic ABMR may serve as a bridge in establishing treatment strategies to improve graft outcomes. In this in-depth review, we focus on the pathophysiology and characteristics of chronic ABMR along with the newly revised Banff criteria in 2017. In addition, in terms of chronic ABMR, we identify the reasons for the resistance of current immunosuppressive therapies and look at ongoing research that could play a role in setting better treatment strategies in the future. Finally, we review non-invasive biomarkers as tools to monitor for rejection.

## Introduction

Successful kidney transplantation (KT) provides a better quality of life and survival compared to transplant candidates (Gill et al., 2005; Oniscu et al., 2005; Finkelstein et al., 2012). Graft outcomes have improved in the past decades, and the short-term graft survival rate is over 95% (Lamb et al., 2011; Hart et al., 2019; Poggio et al., 2020a). Despite the improvement, long-term failure is still high, and 10-years graft failure is 49.7% for deceased donor recipients and 34.1% for living donor recipients (Hart et al., 2019). In a prospective study of indication biopsies, 64% of graft failure was attributed to rejection and all had evidence of antibody-mediated rejection (ABMR) (Sellarés et al., 2012).

T cell-mediated rejection (TCMR) is the most frequent rejection within one year after transplantation but decreases noticeably over time (Sellarés et al., 2012; Halloran et al., 2015). T cell-mediated rejection may be treatable without leading to graft failure (Einecke et al., 2009; Halloran et al., 2010; Sellarés et al., 2012; Halloran et al., 2015). However, a recent study showed that TCMR was associated with early allograft chronicity and progressive renal dysfunction (Hoffman et al., 2019). The 2017 Banff Conference included inflammation in areas of atrophy-fibrosis (i-IFTA) ≥ 2 as part of the diagnostic criteria for chronic active TCMR (Haas et al., 2018). Meanwhile, recent studies suggest that i-IFTA should be reconsidered in the diagnosis of chronic active TCMR as the relationship remains uncertain (Halloran et al., 2019; Helgeson et al., 2020). In the future, the classification of TCMR and its impact on graft outcome will need to be re-examined as further research results become available.

In contrast, ABMR, particularly chronic ABMR, is the leading cause of graft failure caused by rejection (Einecke et al., 2009; Sellarés et al., 2012). Although the pathogenesis of ABMR has not yet been fully identified, significant advances have recently been made in diagnosing and monitoring rejection of renal allografts. The 2017 Banff Conference revised the diagnostic criterion for ABMR, especially chronic active ABMR (Haas et al., 2018). Various clinical trials have been conducted to find new targets or treatment strategies to improve long-term graft survival in recipients with ABMR. Therefore, this review will describe the immune mechanisms from the perspective of chronic ABMR and the characteristics and risk factors of chronic ABMR. Effective strategies for treatment and monitoring methods of chronic ABMR will also be discussed.

### Immune Mechanisms of Antibody-Mediated Rejection

Recognition of alloantigens on the allograft by recipient T cells is the first step of allograft rejection (Becker et al., 2016). This process occurs via direct or indirect allorecognition. In the direct allorecognition pathway, recipient T cells recognize alloantigen on the surface of donor antigen presenting cells (APC) (Becker et al., 2016). Activated CD8 T cells then require the help of activated CD4 T cells to differentiate into cytotoxic effectors (Siu et al., 2018). Direct allorecognition by recipient CD4 T cells is only possible within the first few weeks after transplantation due to the short lifespan of donor APC (Becker et al., 2016; Siu et al., 2018). For this reason, the direct pathway mainly occurs early after transplantation. CD8 T cells can also be activated by semi-direct presentation of intact MHC class I alloantigen on recipient dendritic cells by transfer of donor-derived extracellular vesicles (Siu et al., 2018). The dendritic cells can simultaneously process MHC class I allopeptide and connect to CD8 T cells with the help of CD4 T cells (Siu et al., 2018). In a study by Hughes et al., this “cross-dressing” of recipient dendritic cells occurred early after transplantation and persisted 8 weeks after transplantation in the allograft (Hughes et al., 2020). Thus, allograft rejection by late activation of CD8 T cells can occur even after the donor dendritic cells disappear. In the indirect allorecognition pathway, alloantigens of the graft are processed into peptide fragments by the recipient APC and presented to T cells (Becker et al., 2016; Siu et al., 2018; Leibler et al., 2019). T cells differentiate into T follicular helper T (Tfh) cells following the recognition of the alloantigens on recipient APC (Leibler et al., 2019). The pivotal role of Tfh cells is to provide help to B cells to differentiate into plasma B cells producing high affinity, class-switched antibodies (Wallin, 2018; Laguna-Goya et al., 2019). Circulating Tfh cells from lymph nodes migrating into the blood also play a similar role to Tfh cells in lymph nodes (Wallin, 2018; Louis et al., 2020). B cells activated by antigens undergo differentiation into short-lived plasma cells that secrete antibodies or develop a germinal center (GC) after migrating to B cell follicles with Tfh cells (Leibler et al., 2019). Long-lived plasma cells (LLPCs) and memory B cells are produced in the GC with the help of Tfh cells (Wallin, 2018). The LLPCs are capable of secreting large quantities of antibodies and lasting humoral immunity by interleukin-6 (IL-6) and a proliferation-inducing ligand in bone marrow (Nutt et al., 2015). In LLPC, the surface markers CD19 and CD20 present in other B cell lineages are downregulated (Chong and Sciammas, 2015). The LLPCs without CD20 produce high-affinity antibodies for alloantigen in bone marrow (Ionescu and Urschel, 2019). In contrast, the quiescent memory B cells expressing CD19, CD20, and BAFF-R can rapidly and vigorously differentiate into short-lived PCs secreting antibodies when they are re-exposed to the antigen (Chong and Sciammas, 2015; Ionescu and Urschel, 2019). Acquisition of mutations at the antigen combining site of memory B cell during GC reaction may lead to the formation of dnDSA with unpredicted or multiple HLA specificities (Chong and Sciammas, 2015). Moreover, persistent low-grade activation and differentiation of memory B cells into plasma cells may lead to accumulation of DSA and graft dysfunction (Kometani et al., 2013; Chong and Sciammas, 2015). Amrouche et al. and Caillard et al. reported that preformed DSA with high MFI levels at the time of transplantation and the persistence of preformed DSAs were related to acute or chronic ABMR as well as poor graft survival (Amrouche et al., 2017; Caillard et al., 2017). Preformed DSA that disappeared after transplantation did not significantly affect the prognosis of renal allograft (Caillard et al., 2017). Although the role of memory B cells in chronic ABMR is not yet clear, memory B cells may contribute to the development of chronic ABMR due to preformed DSA.

In the process of antibody production, the interaction between Tfh cells and B cells plays a crucial role, which requires the signals of checkpoint molecules, known as co-stimulatory and co-inhibitory molecules, and cytokines (Leibler et al., 2019). Co-stimulatory molecules and cytokines contribute to activation, differentiation, and function of Tfh cells and B cells (Yan et al., 2017; Leibler et al., 2019). Co-inhibitory molecules modulate the over-activation of Tfh cells (Yan et al., 2017; Leibler et al., 2019). The best studied checkpoint molecules are CD28 and CTLA-4 expressed on T cells and CD80 and CD86 expressed on B cells (Ford et al., 2014; Leibler et al., 2019). Binding of CD28 with CD80/CD86 regulates Tfh cell activation and Tfh-B-cell crosstalk. In contrast, binding of a cytotoxic T-lymphocyte-associated protein 4 (CTLA-4) with CD80/CD86 generates inhibitory signals in T cells (Ford et al., 2014; Leibler et al., 2019). Blocking CD40 on B cells, which upregulates CD80/CD86 by binding of CD40 ligand (CD154) on T cells, can also interfere with antigen-specific GC B cell generation, high affinity class-switching antibody production, and antigen-specific Tfh cell formation (Wallin, 2018; Leibler et al., 2019). Many cytokines are critically involved in the interaction between Tfh cells and B cells. In particular, interleukin-21 (IL-21) produced by Tfh cells maintain the differentiation of Tfh cells and promote the growth, survival, and isotype switching of B cells (Yan et al., 2017). Interleukin-6 (IL-6) plays an important role in the differentiation and function of Tfh cells, the differentiation of B cells into PC or memory B cells, and long-term survival of PC (Yan et al., 2017; Leibler et al., 2019). The BAFF molecule, also known as B-lymphocyte stimulator (BLyS), is a critical factor in B-cell survival and proliferation and binds to BAFF-R and BCMA predominantly expressed on B cells (Banham et al., 2018; Leibler et al., 2019).

Donor-specific anti-HLA antibodies (DSA) trigger ABMR by three mechanisms: complement-dependent cytotoxicity, antibody-dependent cellular toxicity, and direct endothelial injury by DSA (Zhang, 2018). Complement-dependent cytotoxicity is key to active ABMR (Bhalla et al., 2020). Binding of DSA to alloantigen, particularly mismatched class I MHC molecules, activates the classical complement pathway, leading to the release of anaphylatoxins and the recruitment of inflammatory cells, and the formation of the membrane attack complex (Zhang, 2018). Subsequently, this pathway causes tissue injury. In addition, C4d, a degradation product of complement, binds to endothelial basement membrane and is an *in-situ* marker of complement activation on allograft renal biopsies (Rajalingam, 2016; Zhang, 2018). Innate immune cells carrying Fc receptors are involved in antibody-dependent cellular toxicity (Hirohashi et al., 2012; Zhang, 2018). Natural killer cells and macrophages bind to the Fc region of DSAs, triggering degranulation, cell lysis, and phagocytosis (Hidalgo et al., 2010; Lin et al., 2016; Zhang, 2018). Direct binding of DSA to antigens expressed on allograft endothelial cells can also lead to endothelial activation and proliferation (Becker et al., 2016; Zhang, 2018). In chronic ABMR, the latter two complement independent mechanisms appear to play important roles, especially considering that C4d-negative ABMR is relatively common, and eculizumab, which blocks complement factor C5, does not attenuate antibody-mediated damage in chronic ABMR, and NK cell transcript expression is associated with late ABMR rather than early ABMR (Einecke et al., 2009; Gaston et al., 2010; Hidalgo et al., 2012; Haas et al., 2014; Kulkarni et al., 2017).

Missing-self recognition by recipient natural killer cells can also damage allogenic target cells. An inhibitory killer cell immunoglobulin-like receptors (KIRs) expressed on natural killer cells recognize the absence of relevant HLA class I ligands on the allograft, mitigates inhibitory signals, and eliminate the target cells (Rajalingam, 2016). Recently, Koenig et al. reported that missing-self-induced natural killer cell activation could trigger antibody-independent microvascular inflammation of graft and be associated with chronic vascular rejection and poor graft survival (Koenig et al., 2019). Moreover, the combination of missing-self was synergistically deleterious for graft survival in patients with chronic ABMR with DSA (Koenig et al., 2021). However, Yagisawa et al. described that acute ABMR was provoked by natural killer cells and DSA, whereas chronic ABMR was caused by high DSA titers in the absence of natural killer cells activation (Yagisawa et al., 2019). The role of natural killer cells requires further investigation.

### Banff Classification of Antibody-Mediated Rejection

The Banff classification of chronic ABMR is continuously evolving. The Banff 2013 accepted C4d-negative ABMR and antibody mediated intimal arteritis in the diagnosis of ABMR, and chronic active ABMR was revised to chronic ABMR with significant microvascular inflammation (Haas et al., 2014; Haas, 2018a). Moreover, C4d staining in peritubular capillaries was recognized to be associated with the presence of DSA and the equivalence of C4d and DSA on graft outcome was proven (Gaston et al., 2010; Böhmig et al., 2016). Along with molecular classifiers (Sellarés et al., 2013; Loupy et al., 2014), C4d has shown the potential to replace DSA and improve the ability to diagnose ABMR. The Banff 2017 accepted C4d and molecular classifiers as surrogate markers for DSA (Haas et al., 2018). In addition, the Banff 2017 removed the word “acute” from “acute/active ABMR” and left “active” indicating ongoing disease activity because the word “acute” in acute/active ABMR could be accepted as simply referring to evidence of a current or recent event (Haas et al., 2018). The diagnostic criteria for chronic active ABMR was also revised to require all of three criteria: 1) morphological evidence of chronic tissue injury (including at least one of transplant glomerulopathy, severe peritubular capillary basement membrane multilayering, or new-onset arterial intima fibrosis), 2) current/recent antibody interactions with vascular endothelium (including one or more of linear C4d staining of peritubular capillaries, at least moderate microvascular inflammation, or increased expression of gene transcript/classifiers in biopsy tissue strongly associated with ABMR), and 3) serological evidence of DSA (including one or more of DSA to HLA or other non-HLA antigens, C4d staining, or expression of transcript/classifiers) (Haas et al., 2018). Chronic ABMR is diagnosed when there is morphological evidence of chronic tissue injury but there is no evidence of active antibody interaction with the endothelium.

### Phenotype of Antibody-Mediated Rejection

The Banff classification has been continuously evolving toward an accurate diagnosis of ABMR. However, a classification schema based on histological features has limitations in reflecting the complex clinical features of ABMR. The timing of allograft rejection, the presence of DSA, and the time of occurrence of DSA all have a significant influence on the treatment response and prognosis of the allograft (Böhmig et al., 2002; Einecke et al., 2009; Gaston et al., 2010; Loupy et al., 2011; Aubert et al., 2017; Haas et al., 2017). Thus, understanding the underlying pathological processes is required to develop effective therapeutic strategies. The 2019 Transplantation Society Working Group considered the timing of presentation and type of DSA (preexisting or *de novo*) with the histological classification: early posttransplant (<30 days) active ABMR, late (>30 days) posttransplant ABMR with preexisting DSA, and late (>30 days) ABMR associated with *de novo* DSA (dnDSA) (Schinstock et al., 2020).

Early posttransplant active ABMR presents with an abrupt allograft dysfunction in patients with DSA or immunologic amnestic response to alloantigens at the time of transplantation (Haas et al., 2017; Schinstock et al., 2020). It is usually C4d positive and has DSAs against either HLA class I or class II (Gloor et al., 2010; Haas et al., 2017). Fortunately, allograft function can be restored through rapid diagnosis and treatment, and the histological features of active ABMR are often completely resolved (Schinstock et al., 2020). Furthermore, early posttransplant active ABMR is uncommon due to advances in crossmatch technologies (Becker et al., 2016; Schinstock et al., 2020).

Late posttransplant ABMR progresses slowly and is commonly identified on a surveillance biopsy or a for-cause biopsy for minor allograft dysfunction (Gloor et al., 2007; Wiebe et al., 2012; Aubert et al., 2017). Depending on the timing of the biopsy, subclinical, active, or chronic active ABMR is diagnosed (Haas, 2018b; Schinstock et al., 2020). Although the allograft biopsy shows subclinical ABMR, it can proceed to transplant glomerulopathy (TG) or allograft failure (Moreso et al., 2012). In one study, allografts with subclinical ABMR developed dysfunction at a median of 24 (range 6–64) months (Wiebe et al., 2015). ABMR with dnDSA usually occurs 3 months or later after transplantation and the majority are associated with anti-HLA class II (Haas et al., 2017; Schinstock et al., 2020). In a study by Aubert et al., ABMR with dnDSA was diagnosed at a median of 1,437 (range, 437–3,127) days after transplantation, whereas ABMR with the preexisting DSA was diagnosed at a median of 85 (range 17–369) days (Aubert et al., 2017). Wiebe et al. reported that dnDSA developed in nonsensitized recipients at a median 49 (range 6–143) months, with rates 2, 10, 27% after 1, 5, and 12 years (Wiebe et al., 2015). De novo DSA is a significant risk factor for TG and allograft failure (Aubert et al., 2017; Haas et al., 2017). Upon dnDSA detection, 69% had class II dnDSA, 17% had both class I and class II dnDSA, and 14% had class I dnDSA (Wiebe et al., 2015). Immunoglobulin G (IgG) subclasses are also related to the phenotypes of ABMR. Acute ABMR is mainly accompanied by IgG3 DSA, whereas subclinical ABMR is accompanied by IgG4 (Lefaucheur et al., 2016). Subclass IgG3 is associated with a shorter time to graft failure and C4d deposition in allograft peritubular capillaries. Subclass IgG4 is associated with a slower progression to graft failure and a lower incidence of C4d deposition (Lefaucheur et al., 2016). But IgG4 is associated with transplant glomerulopathy and interstitial fibrosis/tubular atrophy. Furthermore, ABMR with dnDSA is associated with more expression of IFN*γ*-inducible, natural killer cell, and T cell transcripts than ABMR with preformed DSA on microarray analysis (Aubert et al., 2017). T cell-mediated rejection may be more often encountered in ABMR with dnDSA than ABMR with preformed DSA (Haas et al., 2017). These distinct immunologic features may explain why the current therapeutic strategies have shown different responses in recipients with ABMR. Therefore, understanding of ABMR phenotypes might provide more specific therapeutic approach for chronic active ABMR.

### Chronic Active Antibody-Mediated Rejection

Chronic active ABMR is not a distinct disease different from active ABMR, but an intermediate stage in the progression of morphologic lesions of active to chronic ABMR. Chronic active ABMR mainly shows the phenotype of late posttransplant ABMR with dnDSA, particularly class II dnDSA. However, chronic active ABMR can develop in recipients with preformed DSA. In a study by Haas et al., chronic active ABMR was diagnosed in 60% of recipients with dnDSA and 30% of recipients with preformed DSA (Haas et al., 2017). In chronic active ABMR, DSA is mainly non-complement binding IgG2 and IgG4 (Zhang, 2018). Lack of C4d staining is relatively common in chronic active ABMR (Gloor et al., 2007; Becker et al., 2016). These findings suggest that chronic active ABMR can occur in the absence of complement activation. Transplant glomerulopathy (TG) characterized by glomerular capillary walls with double contours is not pathognomonic for chronic active ABMR, but it is a major finding of chronic active ABMR (Gloor et al., 2007). The progression of TG is indolent, but TG can occur early and when it does it is associated with poor graft survival (Gloor et al., 2007; Wavamunno et al., 2007). In a study by Wavamunno et al., ultrastructural changes of TG were detected at 39 days after transplantation and changes on light microscopy occurred at 2.3 years after transplantation (Wavamunno et al., 2007). Subclinical TG, as well as TG detected on for cause biopsies, is associated with a high risk of allograft failure (Gloor et al., 2007). The 10-years death-censored graft survival was 57.1% in allograft with TG (Wavamunno et al., 2007). Thus, therapeutic strategies that primarily eliminate Class I DSA, particularly IgG1 and IgG3 subclasses, or drugs that block complement may be less effective for chronic active ABMR. Based on pathophysiologic mechanisms of chronic active ABMR, an early and appropriate therapeutic approach may prevent allograft failure.

### Predictors of Poor Outcome Related to Chronic Antibody-Mediated Rejection

De no DSA is a major cause of chronic ABMR and is associated with poor allograft outcomes. In nonsensitized patients, dnDSA was detected in 11 and 20% at 1 year and 5 years after transplantation (Everly et al., 2013). In a study by Schinstock et al., the mean time to dnDSA detection was 1.8 years after transplant, and only 3.2% of patients developed dnDSA within 1 year after transplant (Schinstock et al., 2017). After dnDSA development, graft loss occurred in 24% within 3 years (Everly et al., 2013). Whereas the incidence of chronic ABMR was 39.5% at 1 and 7-years renal allograft survival was 76% in recipient with high levels of preformed DSA (Amrouche et al., 2017).

Insufficient immunosuppression from nonadherence and reduction of the immunosuppressive agents, cellular rejection, young age, deceased-donor transplant recipients, pretransplant HLA antibodies, HLA mismatch, especially including HLA DQ mismatch, were independent factors for dnDSA formation (Wiebe et al., 2012; Everly et al., 2013; Wiebe and Nickerson, 2013; Gupta et al., 2014). Insufficient suppression of T cells from nonadherence with medications and reduction of the immunosuppressive agents is an important cause of the development of dnDSA (Wiebe et al., 2012; Wiebe and Nickerson, 2013; O’;Leary et al., 2016). Insufficient immunosuppression may be detected by measurement of calcineurin levels. Sapir-Pichhadze et al. demonstrated the relationship between variability in tacrolimus trough blood levels as a surrogate marker of nonadherence and poor graft outcomes such as late allograft rejection, transplant glomerulopathy, and graft failure (Sapir-Pichhadze et al., 2014).

Cellular rejection may be a predictor of dnDSA formation (Wiebe et al., 2012; Cherukuri et al., 2019). Inflamed renal microcirculation during TCMR may contribute to the development of dnDSA (Wiebe and Nickerson, 2013). In one study, at the time of dnDSA detection, 20.0% of biopsies showed acute cellular rejection, but the prevalence of acute cell rejection did not change 1 year after dnDSA detection. In contrast, acute active ABMR increased from 25.0 to 52.9% and chronic AMBR from 7.5 to 38.2% one year after detection of dnDSA (Schinstock et al., 2017). Young age may increase the risk of dnDSA formation due to more robust immune system or greater nonadherence (Oʼ;Leary et al., 2016). Mismatching at HLA A, B, or DR loci are risks for dnDSA, but mismatching at HLA DQ is currently emerging as a greater cause of dnDSA (Willicombe et al., 2012). Discrepancies at both DR and DQ increase the risk for developing *de novo* class II DSA compared to discrepancy at either DR or DQ alone (Willicombe et al., 2012). Moreover, DQ DSA has been associated with risk not only for ABMR, but also transplant glomerulopathy, and graft failure (Willicombe et al., 2012). The advances in tissue typing technology have enabled HLA matching at the epitope level (Lim et al., 2018). Epitopes composed of HLA are polymorphic amino acid residues recognized by B cells, leading to dnDSA formation, TG, and graft loss (Lim et al., 2018; Sypek et al., 2018). HLA-DR/DQ eplet mismatch and tacrolimus less than 5 ng/mL increase the risk of the development of dnDSA (Wiebe et al., 2017). Importantly, however, not all DSAs may be harmful to the graft. In the absence of ABMR, neither preformed DSA or dnDSA affect graft survival (Parajuli et al., 2019). Therefore, tests for correlation with complement as well as DSA strength and specificity to define the pathogenicity of DSA should be considered, but these tests also need to be verified in chronic active ABMR.

In addition to anti-HLA antibodies, non-HLA antibodies are associated with rejection and poor graft survival and damage allograft independently or more seriously in conjunction with anti-HLA antibodies (Zhang and Reed, 2016). Non-HLA antibodies are classified into two main categories: alloantibodies directed against polymorphic antigens, such as major histocompatibility complex class I-related chain A (MICA), and antibodies recognizing self-antigens, for example, anti-angiotensin II type 1 receptor antibodies (anti-AT1R), anti-endothelin-1 type A receptor antibodies, poly-reactive antibodies, and anti-LG3 antibodies (Delville et al., 2016; Zhang and Reed, 2016). Reindl-Schwaighofer et al. demonstrated that non-HLA antibodies against genetically mismatched peptides identified in patients with chronic ABMR were associated with an increased risk of graft loss independently of HLA incompatibility (Reindl-Schwaighofer et al., 2019). Autoantibodies may be detected pretransplantation or develop *de novo* posttransplantation (Filippone and Farber, 2015; Zhang and Reed, 2016). However, in a study by Sun et al., only *de novo* non-HLA antibodies, not preformed non-HLA antibodies, were associated with rejection, especially C4d negative ABMR (Sun et al., 2011). These finding suggests that allograft injury by non-HLA antibody may be caused by complement-independent mechanisms. However, the immunologic phenotypes of non-HLA antibody-mediated rejection remain largely unknown. Much research is also needed on the relationship between non-HLA antibodies and chronic active ABMR.

The presence of C4d, a footprint of alloantibody-mediated classical complement activation, is associated with a high risk of graft failure in chronic active ABMR (Issa et al., 2008; Lesage et al., 2015). However, C4d staining is not a sensitive indicator of chronic ABMR and chronic active ABMR is more related to complement-independent mechanisms of antibody-mediated injury (Loupy et al., 2011; Böhmig et al., 2016). The C1q assay is a test detecting antibodies fixing with complement (Böhmig et al., 2016). In a study by Calp-Inal et al., C1q binding DSA was significantly associated with acute and chronic AMR as well as poor graft survival compared with C1q negative DSA and DSA negative patients (Calp-Inal et al., 2016). Other studies demonstrated that the C1q assay could identify patients at risk for TG and graft loss (Yabu et al., 2011; Guidicelli et al., 2016; Bailly et al., 2018). However, the C1q binding activity in recipients with ABMR is affected by antibody strength (Yell et al., 2015), which may suggest that the C1q assay is not necessary if the antibody strength test is assessed. Sicard et al. suggested that the C3d binding DSA assay was better associated with the risk of graft loss than the C1q binding DSA (Sicard et al., 2015). In other studies, glomerular C3 deposition was associated with the risk of graft failure in patients with TG and glomerular C5b9 deposition was associated with early onset of glomerular basement membrane duplication and poor graft survival (Goutaudier et al., 2019; Panzer et al., 2019). More studies are needed to elucidate the role of complement binding DSAs in predicting ABMR, including chronic active ABMR, and graft loss.

### Treatment of Chronic Antibody-Mediated Rejection

Most studies of chronic ABMR treatment were small, heterogenous, and retrospective trials (Table 1). To date, there are no approved effective treatments for chronic active ABMR, and it remains a major challenge in the field of transplantation. One small retrospective study from Taiwan showed that aggressive treatment which included double-filtration plasmapheresis and one or more of the followings: rituximab, intravenous immunoglobulin (IVIG), antithymocyte globulin, bortezomib, or methylprednisolone pulse therapy, especially at the early stage of chronic active ABMR, was associated with better survival than those who received supportive treatment alone (Chiu et al., 2020). However, the incidence of adverse events was higher in the aggressive treatment group (Chiu et al., 2020). It may be necessary to provide personalized treatment according to the timing and cause of chronic active ABMR. The next section will deal, further, with the treatment of current ABMR, identify their effects and problems in chronic active ABMR, and discuss future treatment directions in chronic active ABMR.

**TABLE 1:** Studies for therapy of chronic antibody-mediated rejection.

Tx	Study design (no. of Ptx)	Diagnostic criteria	Treatment	ISAs	Time after CAMR	Graft loss	Graft function	Ref
PE and IVIG	Retrospective cohort study (123)	Chronic active ABMR	Steroids, IVIG, PE, RTX, or ATG vs no Tx	All ptx received CNIs	4.3 years	Steroids + IVIG	NR	Redfield et al. (2016)
		−BANFF 2013				−HR 0.38 for graft loss (in unadjusted analysis)		
						No additional benefit of rituximab or ATG to CS/IVIG		
	Retrospective cohort study (69)	Chronic active ABMR	IVIG + CS	Variable	6.3 years	Graft survival at 1, 3 and 5 years after chronic active ABMR diagnosis	ΔeGFR	Sablik et al., (2019)
		-BANFF classification				-Responders: 100, 75 and 59%	−9.8/yr prior to tx	
						-Non-responders: 89, 57 and 20%	−6.3/yr after tx (*p* < 0.05)	
RTX	Prospective cohort study (20 pediatric ptx)	Chronic ABMR	All ptx received IVIG + RTX	All ptx received CS + MMF + TAC or CsA	2 years	20%	ΔeGFR from −7.6 during 6 months prior to tx to −2.1 during 6months after tx (*p* < 0.05)	Billing et al. (2012)
		-BANFF 2005						
	Retrospective cohort study (59)	Chronic ABMR	IVIG + RTX + CS vs CS	All ptx received CNIs	>2 years	IVIG + RTX + CS	ΔeGFR 0.2/month in RTX group (*p* < 0.05 compared to the pre-6 months ΔeGFR)	Chung et al. (2014)
		-BANFF 2005				−HR 0.24 for graft loss	ΔeGFR −1.4/month in control group (*p* = NS compared to the pre-6 months ΔeGFR)	
	Retrospective cohort study (21)	Chronic ABMR with TG	IVIG + CS + RTX vs no IVIG + RTX	CNIs	2 years	53% in RTX group	NR	Bachelet et al. (2015)
		-BANFF 2013		95% in RTX group		60% in control group		
				80% in control group				
	Retrospective cohort study (62)	Chronic active ABMR with TG	IVIG + PE + RTX vs no Tx	Variable	27 months	NS	NS	Piñeiro et al. (2018)
		-BANFF 2017						
	Placebo-controlled RCT (25)	Chronic ABMR: TG ± C4d in ptc; anti-HLA DSA	IVIG + RTX vs placebo	All ptx received TAC + MMF	1 year	8% in RTX group	ΔeGFR	Moreso et al. (2018)
						8% in placebo group	−4.2 in RTX group	
							−6.6 in placebo group (*p* = NS)	
Bortezomib	Prospective cohort study (30)	Early acute ABMR (13 ptx) and late acute ABMR (17 ptx)	All pts received PE + RTX + bortezomib	All ptx received TAC + MMF	7 months	10% in early ABMR	eGFR from 40 prior to tx to 66 after tx in early ABMR (*p* < 0.05)	Walsh et al. (2011)
		-BANFF 2007				20% in late ABMR	eGFR from 27 prior to tx to 37 after tx in late ABMR (*p* < 0.05)	
	Placebo-controlled RCT (44)	Late ABMR	Bortezomib vs placebo	All ptx received CS + MMF + TAC or CsA	2 years	19% in bortezomib group	eGFR slope	Eskandary et al. (2018b)
		BANFF 2013 (28 ptx: chronic active ABMR)				4% in placebo group (*p* = NS)	−4.7/year in bortezobmib group −5.2/year in placebo group (*p* = NS)	
Eculizumab	Nonblinded RCT (20)	Chronic ABMR: DSA >MFI 1100; 20% reduction in eGFR; no severe fibrosis	Eculizumab vs control	CNIs or rapamycin	1 year	NR	NS	Kulkarni et al. (2017)
C1 inhibitor	Prospective phase 1 study (10)	Late ABMR	All ptx received C-INH (BIVV009)	All ptx received CS + MMF + CNIs (9 ptx – TAC, 1 ptx - CsA)	50 days	NR	Stable	Eskandary et al. (2018a)
		-BANFF 2013 (9 ptx: chronic active ABMR)						
IL-6 Tocilizumab	Prospective cohort study (36)	Chronic ABMR with TG - BANFF 2013	All ptx received tocilizumab	All ptx received CS + MMF + TAC	3.3 years	11.1%	Stable	Choi et al. (2017)
Clazakizumab	Randomized, placebo controlled, parallel-group phase 2 trial (20)	Late active or chronic active ABMR ≥ 365 days post-transplantation with a molecular pattern of ABMR	Clazakizumab vs palcebo	18 ptx received CNIs or mTOR inhibitor-based triple therapy, 2 ptx received dual therapy without steroids	52 weeks	1 ptx at 3 months after last visit	eGFR slope −0.96/month in clazakizumab group	Doberer et al. (2020)
							−2.43/month in placebo group (*p* < 0.05)	
							Improvement of eGFR slope in pts switched from placebo to clazakizumab (*p* < 0.05)	

eGFR is reported as mL/min/1.73 m^2^.

ATG, rabbit antithymocyte globulin; CAMR, chronic antibody-mediated rejection; CNIs, calcineurin inhibitors; CS, corticosteroid; CsA, cyclosporin A; DSA, donor-specific antibodies; eGFR, estimated glomerular filtration rate; HR, hazard ratio; ISA, immunosuppressive agent; IVIG, intravenous immunoglobulin; MMF, mycophenolate mofetil; NR, not reported; NS, non-significant; PE, plasma exchange; ptc, peritubular capillary; Ptx, patient; RCT, randomized controlled trial; Ref., reference; RTX, rituximab; TAC, tacrolimus; TG, transplant glomerulopathy; Tx, treatment; Δ, change in.

#### Conventional Immunosuppressive Therapy

The differentiation of B cells into LLPCs and the production of high-affinity antibodies are completed with the help of Tfh cells in the germinal center reaction (Wallin, 2018). Effective Tfh cell suppression is crucial to prevent the development of dnDSA. However, conventional immunosuppressive therapies are not thoroughly effective at limiting Tfh cells. Cano-Romero et al. evaluated the effects of induction therapy on circulating Tfh (Cano-Romero et al., 2019). Thymoglobulin significantly depleted circulating Tfh but circulating Tfh recovered within 6 months. Basiliximab did not affect the elimination of circulating Tfh (Cano-Romero et al., 2019). Moreover, in a study by Danger et al., thymoglobulin and calcineurin inhibitors (CNIs) were associated with a decreased total circulating Tfh cells but activated circulating Tfh cells increased (Danger et al., 2019). Corticosteroids did not affect circulating Tfh cell distribution (Danger et al., 2019). Although Tfh cell is dependent on nuclear factor of activated T (NFAT) signaling, Tfh cells appear only to be partially inhibited by CNIs and are still able to help B cells to differentiate into plasma cell producing antibodies (Laguna-Goya et al., 2019). In one study, tacrolimus had a minimal inhibitory effect on Tfh cell generation and could only partially prevented Tfh cell activation *in vitro* (de Graav et al., 2017). Cyclosporin has a 2.7 times higher incidence of dnDSA development than tacrolimus, suggesting a weaker effect on Tfh cells than tacrolimus (Wiebe et al., 2017). Sirolimus, an mTOR inhibitor, may suppress the number of circulating Tfh cells more efficiently than tacrolimus, but sirolimus is more likely to be associated with the development of dnDSA development than tacrolimus (Laguna-Goya et al., 2019). However, subtherapeutic immunosuppression from nonadherence or reduction of immunosuppressive agents is the main cause of chronic active ABMR although rejection reactions are not fully controlled by the conventional immunosuppressive therapies. Therefore, optimizing baseline immunosuppression and enhancing drug compliance should be a priority.

#### Plasma Exchange and Intravenous Immunoglobulin

The mainstays of active ABMR treatment are plasma exchange (PE) and IVIG (Schinstock et al., 2020). However, studies for the effects of PE and IVIG have not supported their use in patients with chronic active ABMR. Although plasma exchange can reduce levels of plasma antibodies and graft injury, it is a transient effect (Ionescu and Urschel, 2019). Plasma exchange may require a combination of treatments that inhibit the formation of dnDSA, particularly class II dnDSA, by newly matured plasma cells and long-lived plasma cells (Velidedeoglu et al., 2018; Ionescu and Urschel, 2019).

The effects of IVIG appear to be on both innate and adaptive immune systems. It can suppress various innate immune cells such as dendritic cells, monocytes, macrophages, neutrophils, and NK cells. It can also neutralize the active complement component and regulate B cell function, plasma cells, Treg cells and effector T cells, and proliferative cytokines (Galeotti et al., 2017). Cooper et al. reported that high-dose IVIG eliminated class I antibodies more than class II antibodies and did not link to stabilization of graft function in recipients with chronic graft dysfunction, including chronic active ABMR (Cooper et al., 2014). Whereas in a study by Redfield et al., IVIG and steroids were associated with better graft survival in 123 patients with chronic active ABMR (Redfield et al., 2016). A recently published study described that IVIG and methylprednisolone could reduce the loss of renal function decline in more than 60% of patients with chronic active ABMR with a progressive decline in eGFR (Sablik et al., 2019). However, more research is needed to define the effects of IVIG in chronic active ABMR.

#### Anti-CD20 Monoclonal Antibody

Rituximab, a chimeric monoclonal antibody against CD20, eliminates B cells and memory B cells, which leads to depleting allospecific antibodies and anamnestic response (Zhang, 2018). Whereas LLPCs without CD20 are resistant to rituximab and continuously produce alloantibodies (Ionescu and Urschel, 2019). In general, rituximab does not appear to efficiently improve the results of ABMR, although it may have some benefits in active ABMR (Roberts et al., 2012; Macklin et al., 2017). In a multicenter randomized controlled trial for acute ABMR (RITUX-ERAH trial), 38 patients receiving rituximab or placebo were evaluated 1 year after transplantation (Sautenet et al., 2016). The study did not demonstrate additional effects of rituximab in combination with PE, IVIG, and corticosteroids for acute ABMR (Sautenet et al., 2016). Moreover, infectious complications and gastrointestinal disorders occurred more frequently in patients receiving rituximab than in placebo (Sautenet et al., 2016). In the 7-years outcomes of the RITUX-ERAH trial, there was no long-term benefit of rituximab (Bailly et al., 2020).

Chung et al. reported that the use of IVIG and rituximab increased graft survival in patients with chronic active AMBR (Chung et al., 2014). However, most studies evaluating the effects of rituximab on chronic ABMR or chronic active ABMR failed to demonstrate the efficacy of rituximab on graft outcomes. Piñeiro et al. retrospectively evaluated data of 62 patients with chronic active ABMR. Treatment with rituximab, IVIG, and PE was not associated with the improvement of graft survival and increased the incidence of severe infectious complications compared to the control (Piñeiro et al., 2018). Bachelet et al. retrospectively compared 21 patients receiving two doses of rituximab, four doses of IVIG, and corticosteroids with the untreated control group of 10 patients (Bachelet et al., 2015). At 24 months post-biopsy, graft survival was 47 and 40% in the treated and the untreated group (*p* = 0.69) and the incidence of adverse events were higher in the treated group (Bachelet et al., 2015). Recently, in a multicenter, randomized placebo-controlled trial, the efficacy and safety of rituximab combined with IVIG were evaluated in patients with chronic ABMR, including chronic active ABMR. Among 25 patients, 13 patients received IVIG (4 doses of 0.5 g/kg) and rituximab (375 mg/m^2^). There were no significant differences between the treatment and placebo groups in the estimated GFR, proteinuria, 1-year Banff score, and DSA levels (Moreso et al., 2018). Whereas, in a prospective study of 20 pediatric patients with chronic ABMR, treatment consisting of IVIG and rituximab was associated with improvement or stabilization of eGFR, and this response lasted for 24 months. The presence of TG significantly lowered the effectiveness of IVIG and rituximab (Billing et al., 2012). In addition, Kahwaji et al. reported that patients with microvascular inflammation might benefit from IVIG and rituximab treatment in the presence of TG (Kahwaji et al., 2014). These results suggest that the treatment may be effective in the early stages of chronic active ABMR. However, the effect of rituximab in chronic active ABMR cannot be defined because IVIG and rituximab were administered together in previous studies. In addition, since the studies published so far are heterogeneous in drug dosage and combination, the number of treatment cycles, and patients, more studies for rituximab are needed.

Obinutuzumab is a type 2 anti-CD20 antibody and may deplete B cells more than rituximab (Ionescu and Urschel, 2019; Redfield et al., 2019). Recently, Redfield et al. reported that obinutuzumab combined with IVIG potently depleted B cells in hypersensitized patients with ESRD awaiting transplantation. However, there was no clinically meaningful reduction in anti-HLA antibodies (Redfield et al., 2019). The relevance of these findings for the use of obinutuzumab for the treatment of chronic AMR is unclear.

#### Proteasome Inhibitor

Bortezomib is the selective inhibitor of the 26S proteasome and induces cell death of short- and long-lived plasma cells by accumulating unfolded proteins (Neubert et al., 2008). It was thought to lead to the suppression of the production of dnDSA. The Mayo group showed that bortezomib eliminated DSA-producing plasma cells and PE could enhance DSA removal in sensitized renal transplant recipients (Diwan et al., 2011). In another study, combination therapy with bortezomib, rituximab, and PE reduced DSA levels, and it was associated with improved GFR in patients with early or late acute ABMR (Walsh et al., 2011). Eskandary et al. conducted a randomized, placebo-controlled trial with bortezomib in 44 patients with late ABMR (BORTEJECT trial) (Eskandary et al., 2018b). Bortezomib was administered in two cycles of four doses each to 21 patients. The study failed to demonstrate the efficacy of bortezomib in the prevention of GFR loss, graft survival, histologic or molecular rejection phenotypes, and reduction in DSA (Eskandary et al., 2018b). Moreover, bortezomib increased gastrointestinal and hematologic toxicity (Eskandary et al., 2018b). In a study by Philogene et al., bortezomib reduced HLA class I antibody more effectively than class II antibody (Philogene et al., 2014). In addition, Kwun et al. demonstrated that bortezomib-induced plasma cell depletion induced germinal center B cell and Tfh cell expansion in the lymph nodes. This humoral compensation was associated with the generation of new antibody-producing cells and a failure of DSA depletion (Kwun et al., 2017). Furthermore, despite PC depletion, the stability of DSA may occur due to new generation of plasma cells from GC memory B cells, new plasma cells generation due to proliferation within a bone marrow precursor plasma cell population, or reverse differentiation of the LLPC population (Woodle et al., 2017). To date, findings of studies using bortezomib have been inconsistent. This may be caused by the small number of patients enrolled in the studies or differences in drugs used in combination with bortezomib, or because it simply is not effective. A randomized trial is being conducted to evaluate the efficacy of bortezomib in chronic ABMR patients (NCT02201576), and the results may help determine how to use bortezomib in chronic ABMR patients.

Carfilzomib, an irreversible proteasome inhibitor, as opposed to the reversible proteasome inhibitor bortezomib, has a short half-life and less off-target effects, which may have favorable safety compared to bortezomib (Woodle et al., 2020). Ensor et al. reported that carfilzomib in combination with PE and IVIG significantly lowered DSA IgG mean-fluorescence intensity (MFI) and DSA C1q MFI in 10 of 14 lung transplant recipients (Ensor et al., 2017). A recent prospective, nonrandomized trial described that carfilzomib had an acceptable safety and toxicity profile and depleted bone marrow plasma cells and anti-HLA antibodies in highly HLA-sensitized KT candidates (Tremblay et al., 2020). However, antibody levels returned to baseline between days 81 and 141 due to the rebound of the antibody (Tremblay et al., 2020). Woodle et al. suggested that the resistance of plasma cells to carfilzomib may be caused by structural changes of proteasome and immune proteasome formation (Woodle et al., 2020). In addition, carfilzomib-resistant bone marrow plasma cells had low sensitivity to the proteasome inhibitors carfilzomib and bortezomib, whereas they had enhanced sensitivity to an immunoproteasome-specific inhibitor ONX-0914 (Woodle et al., 2020). In a study by Li et al., ONX 0914 depleted the numbers of B and plasma cells and suppressed the production of DSA in rats that received KT (Li et al., 2018). Identifying mechanisms for the resistance of proteasome inhibitors and targeting them may have the potential to improve transplant outcomes.

#### Complement-Based Therapy

The complex of anti-HLA DSA and alloantigen activates the classical pathway of the complement system by binding to C1q (Tatapudi and Montgomery, 2019; Bhalla et al., 2020). The binding of C1q activates C1r and C1s, which subsequently cleaves C4 to C4a and C4b, and eventually, this process produces anaphylatoxins of the C3a and C5a and C5b9 membrane attack complexes (Tatapudi and Montgomery, 2019; Bhalla et al., 2020). Eculizumab is a humanized monoclonal antibody that blocks the cleavage of C5 into C5a and C5b (Bhalla et al., 2020). C1 esterase inhibitor (CI-INH) inhibits the complement proteases C1r and C1r (Tatapudi and Montgomery, 2019). These complement inhibitors have been used in the prevention and treatment of ABMR. Eculizumab showed the potential to have meaningful positive effects on preventing acute ABMR and improving graft survival in living- and deceased-donor KT recipients (Glotz et al., 2019; Marks et al., 2019). However, eculizumab therapy did not prevent chronic ABMR in recipients with persistently high DSA (Cornell et al., 2015). In KT recipients with *de novo* DSA MFI >1100 and a 20% reduction in eGFR during the 12 months prior to enrollment, eculizumab therapy appeared to have the ability to stabilize renal function, but the expression of endothelial cell-associated transcripts predicting acute humoral injury was not reduced (Kulkarni et al., 2017). In an observational retrospective study by Schinstock et al., eculizumab therapy reduced the rate of early active ABMR over a 6.8-years follow-up period in highly sensitive recipients, but this did not lead to a decrease in the rate of chronic active ABMR rate or improvement in graft survival (Schinstock et al., 2019). In studies published so far, eculizumab appears to prevent early ABMR in highly sensitive KT recipients during the period of administration, but this does not seem to lead to changes in long-term outcomes. For that reason, it is thought that C5 blockade by eculizumab may activate upstream complement factors, like the anaphylatoxin C3a, and triggering chronic injury and inflammation. Sublytic levels of the membrane attack complex also might cause endothelial cell activation. Furthermore, complement-independent mechanisms might contribute to endothelial damage (Bhalla et al., 2020).

In previous studies, the use of C1 INH for acute ABMR improved renal function and reduction in TG (Montgomery et al., 2016; Viglietti et al., 2016). In late antibody-mediated kidney allograft rejection, anti-C1s monoclonal antibody BIVV009 inhibited the complement pathway, but there was no change in microcirculation inflammation, gene expression patterns, DSA levels, or kidney function in 5-weeks follow-up biopsies (Eskandary et al., 2018a). However, studies on C1-INH or C1s monoclonal antibodies are too small and heterogenous to define their effectiveness. In the future, more studies are needed to determine their roles in chronic ABMR.

#### IL-6 Inhibitor

IL-6 acts as a key mediator in the innate immune response and adaptive immunity, and the role of IL-6 in allograft rejection has attracted attention. IL-6 stimulates the production of IL-21 in naive T cells, leading to differentiation into Tfh cells with CXCR5, IL-21, and transcription factor Bcl-6. Naive B cells drawn to the germinal center by CXCR5+ Tfh cells differentiate into plasmablasts capable of producing large amounts of IL-6. Together with IL-21, IL-6 induces plasmablasts to differentiate into LLPCs. In addition, IL-6 plays an important role in shaping T cell immunity, especially increasing Tfh cells and inhibiting regulatory T cells. Moreover, the production of IL-6 by binding of DSA to alloantigen on graft endothelium can stimulate intimal proliferation and obliterative vasculopathy, likely resulting in manifestations of chronic ABMR (Jordan et al., 2017). Therefore, blockade of IL-6 may inhibit Tfh cell activity, upregulate Treg cell, and reduce the production of plasmablast and DSA. In addition, it may prevent intimal proliferation and obliterative vasculopathy by IL-6 produced in endothelial cells (Jordan et al., 2017). Recently, Shin et al. showed that tocilizumab, an anti–IL-6 receptor monoclonal antibody, reduced total IgG and IgG1-3 and anti-HLA-total IgG and IgG3 levels in patients with chronic ABMR (Shin et al., 2020). Choi et al. evaluated the efficacy of tocilizumab in 36 patients with chronic ABMR and TG who failed standard of care treatment with IVIg and rituximab with or without plasma exchange. At the time of chronic ABMR diagnosis, 31 (86.1%) of 36 patients had class II HLA-DSAs. Tocilizumab significantly reduced DSAs and stabilized renal function. Graft survival and patient survival rates were 80 and 91% at 6 years (Choi et al., 2017). When tocilizumab was stopped, two patients developed mild ABMR detected on a for-cause biopsy at 1 year, suggesting the possibility of rebound IL-6–IL-6R signaling (Choi et al., 2017). In contrast, Massat et al. evaluated the efficacy of tocilizumab in nine patients with ABMR, including six patients with chronic active ABMR, resistant to apheresis, rituximab, and intravenous immunoglobulins. Tocilizumab had no significant effect on graft survival and renal function at 1-year follow-up (Massat et al., 2020). In another report, the efficacy of tocilizumab therapy was described in 10 patients with chronic active ABMR. The slope of decline in eGFR was unchanged and microvascular inflammation score or Molecular Microscope Diagnostic System (MMDx) ABMR scores was not improved at 1-year follow-up (Kumar et al., 2020). A phase four RCT was designed to evaluate the efficacy of tocilizumab in KT recipients with chronic active ABMR (NCT04561986), but it is not yet recruiting. The results of this study may clarify whether tocilizumab is useful for the treatment of chronic active ABMR in kidney transplant patients.

A similar antibody, clazakizumab, is a humanized monoclonal antibody directed against the IL-6 molecule itself rather than the IL-6 receptor. A randomized, double-blind, placebo-controlled, parallel-group phase two trial was conducted to evaluate the safety and efficacy of clazakizumab in 20 patients with late active or chronic active ABMR >365 days post-transplantation (Doberer et al., 2020). Clazakizumab 25 mg or placebo was administered via subcutaneous injection every 4-weeks for 12 weeks, followed by a 40-weeks open-label extension where all participants received clazakizumab. Clazakizumab therapy significantly reduced DSA and showed a potentially beneficial effect on ABMR activity and renal function. Serious infectious events developed in five patients and complications of diverticular disease occurred in two patients (Doberer et al., 2021). Jordan et al. performed an open-label, single-arm phase I/II study to evaluate the safety and tolerability of clazakizumab in 10 patients with chronic ABMR (NCT 03380377) (Jordan et al., 2020). Clazakizumab 25 mg was administered monthly for 12 months, followed by bimonthly infusions. Five patients underwent 18 months of therapy and two patients were withdrawn due to graft failure and at the request of a patient. After 18 months of follow-up, eight patients showed stabilization of renal function and reductions in mean DSA relative intensity scores (Jordan et al., 2020). Currently, a randomized, double-blind, parallel-group, placebo-controlled, phase three trial using clazakizumab for treatment of chronic active ABMR is underway (NCT03744910). Clazakizumab 12.5 mg or placebo will be administered monthly for up to 65 months in 350 patients with chronic active ABMR.

#### Anti-CD38 Monoclonal Antibody

Daratumumab is a human IgG1 monoclonal antibody targeting CD38, a transmembrane glycoprotein expressed on the surface of many immune cells, including plasma cells, plasmablasts, regulatory T cells, regulatory B cells, and natural killer cells (Krejcik et al., 2016; Kwun et al., 2019). In a recent study, daratumumab therapy significantly reduced anti-HLA DSAs in a non-human primate (NHP) model and a heart/kidney transplant recipient with refractory ABMR and a highly sensitized heart transplant candidate, suggesting the potential of daratumumab as a therapeutic strategy. However, the rapid rebound of DSA and TCMR was observed in NHPs (Kwun et al., 2019). In the study by Krejcik et al., daratumumab activated and expanded cytotoxic T-cells and decreased CD38-expressing immunosuppressive regulatory T and B cells in patients with multiple myeloma (Krejcik et al., 2016). Viola et al. demonstrated that daratumumab therapy decreased the count of total NK cells, but it activated CD38-negative NK cells and enhanced expression of CD80/CD86 T-cell costimulatory molecules on monocytes in patients with multiple myeloma. The T cell population was activated and increased through the binding of CD28 on T cells and CD80/86 on monocytes (Viola et al., 2021). In contrast, Doberer et al. reported the efficacy of daratumumab in a KT recipient with chronic active ABMR and smoldering myeloma. Daratumumab therapy with a 9-months course depleted plasma cells in the bone marrow and blood and NK cells in blood and graft tissue. Donor specific antibodies disappeared in serum. Microcirculation inflammation and molecular AMR activity were improved at a 3-months follow-up biopsy. Subclinical borderline rejection with focal high-grade tubulitis and mild interstitial infiltrates was identified at a follow-up biopsy after 3 months, but no molecular signatures of T-cell–mediated rejection were present and the count of circulating regulatory T cell was not reduced (Doberer et al., 2021). More research is needed on the mechanisms and efficacy of daratumumab, especially in transplant recipients with chronic ABMR.

#### Combination Therapy

The various immunosuppression protocols to date are unable to completely reduce DSA titers, prevent a rebound in antibody production, or significantly improve graft survival, particularly in chronic active ABMR. The 2019 expert consensus of the Transplantation Society working group recommends optimizing conventional immunosuppressive agents and supportive therapies in chronic active ABMR patients with existing DSA or dnDSA because of the poor outcomes and adverse events of IVIG, PE, and/or rituximab (Schinstock et al., 2020). However, chronic active ABMR showing a different phenotype than acute ABMR, namely class II dnDNA, IgG2 and IgG4 subclasses, and activation of the complement-independent pathway, may generally have an incomplete response to current therapy. Additionally, depending on the preformed DSA or dnDSA, different approaches may be needed by focusing on memory B cell or LLPC respectively. However, it is necessary to study whether targeting memory B cells is helpful for the prevention of chronic ABMR with preformed DSA and graft survival.

**FIGURE 1:**
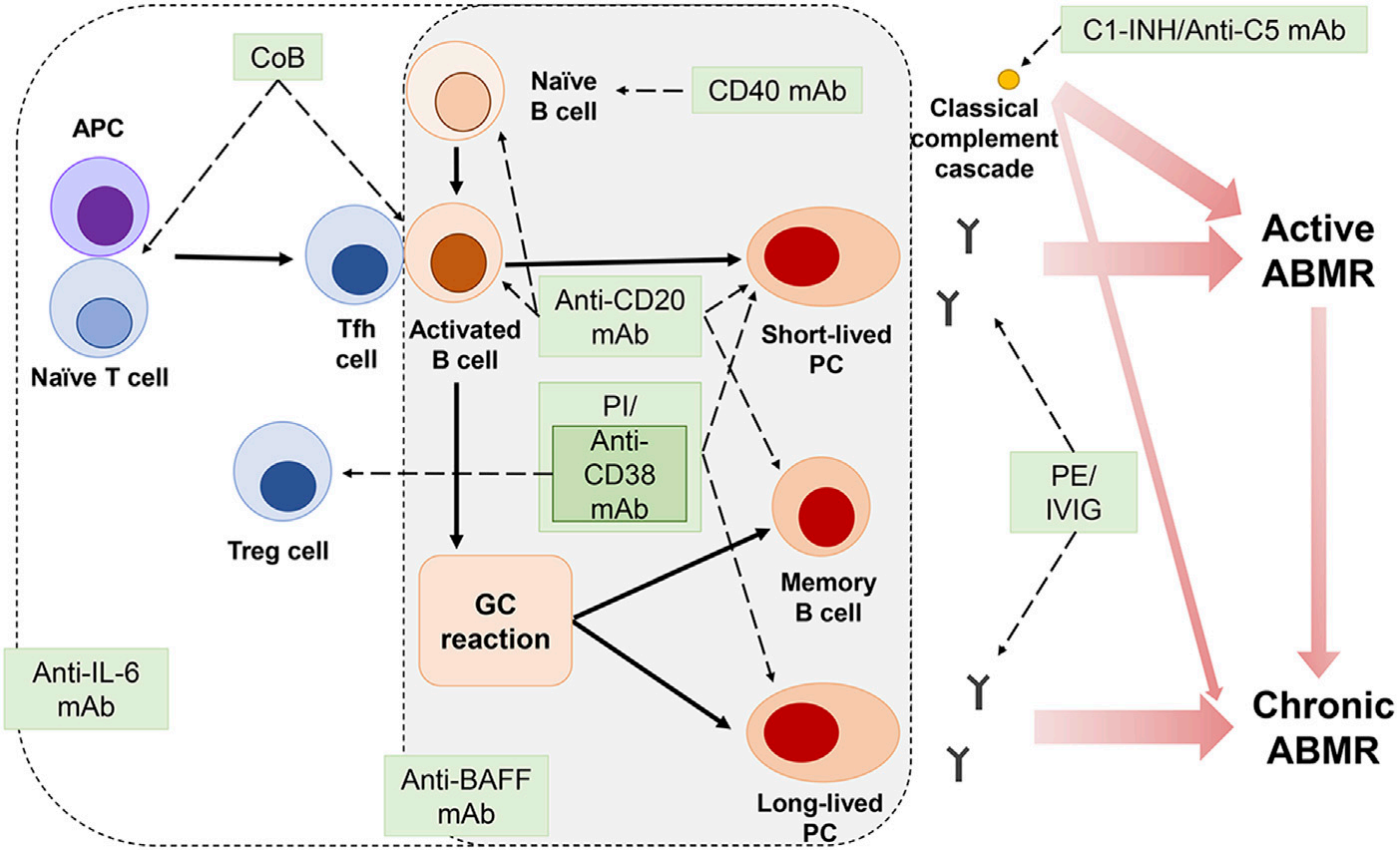
Immune mechanisms of graft rejection and therapeutic targets.Plasma exchange (PE) and intravenous immunoglobulin (IVIG) remove DSAs. Anti-CD20 monoclonal antibody (mAb) eliminates B cells, memory B cells, and short-lived plasma cells (PC). However, PE, IVIG, and anti-CD20 mAb are insufficient to inhibit long-lived PCs that continuously produce antibodies. Although proteasome inhibitors (PI), bortezomib and carfilzomib, can eliminate PCs, the expansion of bortezomib-induced germinal center (GC) B cells and Tfh cells or the appearance of carfilzomib-resistant bone marrow PCs may mitigate the effect of PI. Anti-CD38 mAb depletes PCs, but it can also suppress T regulatory (Treg) cells. Combination strategies using costimulation blockers, anti-interleukin 6 (IL-6), and anti-B-cell-activating factor (BAFF) mAB could further enhance the effectiveness of rejection therapies in blocking antibody production and preventing graft damage by generating synergy of the drugs.

Chronic ABMR relies on allogeneic responses initiated in the GC of the lymph nodes, but antibody production by long-lived plasma cells, the output of the GC response, is resistant to current immunosuppression (Chhabra et al., 2018; Ionescu and Urschel, 2019). The combination of targeting LLPCs and effective B cell depletion may be a promising approach in chronic active ABMR (Figure 1). Leibler et al. demonstrated that belatacept, a human fusion protein combining the extracellular portion of a CTLA-4 immunoglobulin (CTLA-4-Ig), could bind to CD80 and CD86, and prevent the interaction with CD28 in Tfh cells and limit plasmablast differentiation, production of antibody, especially IgG2 and IgG4, and prevent activation of Tfh cells in KT recipients (Leibler et al., 2018). In a study by La Murag et al., selective CD28 blockade sparing CTLA-4 led to superior inhibition of Tfh cells, GC, and DSA responses compared to CTLA-4-Ig (La Muraglia et al., 2020). The non-depleting anti-CD40 antibody, iscalimab, inhibited GC formation and prolonged kidney allograft survival without depleting B cells in NHP (Cordoba et al., 2015). These results suggest the potential therapeutic utility of costimulation blockade in preventing the development of dnDSA and subsequent antibody-mediated graft injury (Cordoba et al., 2015).

Burghuber et al. evaluated the combination of costimulation blockades, with belatacept and anti-CD40 mAb, and bortezomib in a sensitized non-human primate KT model. This combination therapy significantly diminished bone marrow PCs, Tfh cells, and memory B cell proliferation and prolonged graft survival compared to control animals. However, it was associated with significant infectious complications and drug toxicity (Burghuber et al., 2019). In another study, the combination of carfilzomib and belatacept lowered DSAs, bone marrow PCs, and Tfh cells and improved graft survival in highly sensitized NHP KT recipients (Ezekian et al., 2019). Infectious complications or other toxicity were not identified. However, ABMR developed from rebound of the humoral response (Ezekian et al., 2019). In the only human study of this combination, Jain et al. reported that bortezomib therapy with belatacept reduced DSA and reversed ABMR in 6 KT recipients (Jain et al., 2020).

In an NHP model, lulizumab, a selective CD 28 blocker, in combination with carfilzomib significantly reduced DSA, Tfh cells, and proliferating B cells in the lymph nodes in allosensitized NHPs. The combination therapy lowered the ABMR score and prolonged graft survival. Furthermore, lulizumab preserved the Treg cell population during desensitization. However, all desensitized animals eventually developed ABMR and graft failure (Schroder et al., 2020). These studies suggest that the combination of B-cell targeting therapy with co-stimulatory blockade agents has an effect on controlling GC reaction and Tfh cells. However, the development of ABMR, along with rebound of the humoral-response, may indicate the need for sustained suppression of B cell responses. In one animal study, CD28^+^ memory T cells lost CD28 expression after transplantation, which may be associated with resistance to CTLA4-Ig or selective CD 28 blockade (Mathews et al., 2017). Daratumumab, which targetsCD38, may be used in combination with co-stimulatory blockade agents. However, combination therapy with daratumumab may require simultaneous consideration of modulating the activation of cytotoxic T cells and reduction of regulatory T and B cells.

Besides combination therapy targeting B cells, cytokine inhibitors, such as anti- IL-6, anti-IL-21, or anti-BAFF, may be combined with co-stimulatory blockade agents. Zhao et al. suggested the combination of co-stimulatory molecule blockade and IL-6 blockade may lead to better graft survival (Zhao et al., 2012). Another study showed that blockade of the IL-21 receptor had the ability to inhibit the differentiation of B cells to antibody-producing plasmablasts (de Leur et al., 2017). The use of belimumab, a BAFF inhibitor, showed a trend to reduce naïve B cells, circulating plasmablasts, and memory B cells in adult KT recipients (Banham et al., 2018). However, it is not yet possible to determine whether combination therapy with co-stimulatory blockade agents and B-cell targeting therapy or cytokine inhibitor is superior to current immunosuppressive therapy in terms of safety, efficacy, and cost. Combination therapy should be studied more in recipients with ABMR, especially chronic active ABMR.

### Prevention of Chronic Antibody-Mediated Rejection

Chronic ABMR is a major cause of graft failure, but current immunosuppressive therapy is inadequate to treat chronic ABMR, including chronic active ABMR. Prevention of chronic ABMR may be the most effective method at this time. Hence, it is important to manage the factors associated with the occurrence of dnDSA.

Because nonadherence with medication is the leading cause of dnDSA, improving adherence may be the most effective and rational approach for the prevention of chronic ABMR. Related but different, the minimization of the immunosuppressive agents by physicians can lead to subtherapeutic levels, especially of the calcineurin inhibitors. The drug concentration should be kept within an appropriate range, and if the drug is reduced, the drug concentration should be adjusted with monitoring of dnDSA formation. Limiting mismatch of donor-recipient HLA epitopes/eplets may be facilitated by HLAMatchmaker software, which may increase the likelihood of finding compatible matches and minimize sensitization (Wiebe et al., 2013; Zhang and Reed, 2016; Wiebe et al., 2017). Ischemia-reperfusion injury and surgical trauma can cause the release of organ-derived autoantigens and non-HLA antibody formation (Zhang and Reed, 2016). Desensitization treatment for non-HLA antibodies and treatment strategies for AMR with non-HLA antibodies have not been clearly established. It would be necessary to minimize organ damage related to transplant surgery. Cellular rejection is one of the causes of dnDSA formation and active ABMR is at greater risk for chronic ABMR (Wiebe et al., 2012). Early detection and successful treatment of cellular rejection and active ABMR may prevent progression to chronic ABMR.

### Monitoring of Renal Allograft

Allograft biopsy is a gold standard to identify rejection or other histologic lesions (Anglicheau et al., 2016). A renal biopsy is an invasive procedure and may cause severe complications in renal allograft. In native renal biopsy, complications were estimated as hematoma 11%, macroscopic hematuria 3.5%, bleeding requiring blood transfusions 1.6%, and interventions to stop bleeding 0.3%, and death 0.06% (Poggio et al., 2020b). In renal allograft biopsy, complications were gross hematuria 3.5%, perirenal hematoma 2.5%, arterio-venous fistula 7.3%, and major complications requiring invasive procedures were 1% (Schwarz et al., 2005). Overall, biopsy-related complications do not appear to be more common in renal allografts. Surveillance biopsies for all KT recipients were performed in 18% out of US transplant centers, commonly at 3 and 12 months post-transplant (Mehta et al., 2017). A common cause of not performing biopsy was low yield (Mehta et al., 2017). Mengel et al. reported that the incidence of rejection was 7.8 and 21.9%, and borderline changes were 17.9 and 16.0% in protocol biopsies and indication biopsies (Mengel et al., 2007). It would be a burden to clinicians and patients to perform protocol biopsies to check whether allograft rejection has occurred or to monitor the response to therapy. Meanwhile, Sellare´ s et al. showed that a molecular classifier could estimate the probability of ABMR and predict graft failure (Sellarés et al., 2013). The study included KT recipients with a median time of 69 months from transplant to biopsy and most ABMRs were late ABMR more than 1 year after transplantation. The score of the molecular classifier correlated not only with the microvascular inflammation of ABMR, but with ABMR with TG (Sellarés et al., 2013). The MMDx derived from the microarray data also improved the uncertainty for ABMR in graft biopsy (Halloran et al., 2013). However, the MMDx needs graft biopsy specimen for molecular measurements and should be further validated for clinical use in the future.

A non-invasive biomarker can reduce the burden of the graft biopsy to the patient and clinician. The ideal non-invasive biomarker can distinguish the rejection from other renal diseases or transient changes, predict the prognosis of patients and grafts as well as enable patient stratification according to risk, which may provide individualized immunosuppressive drug therapy to patients. To date, in addition to classic biomarkers such as creatinine and proteinuria, various biomarkers have been developed and used in clinical practice or are still under investigation. A non-invasive biomarker for ABMR should be easy to obtain, fast and inexpensive. In addition, it should enable early prediction of the occurrence of ABMR and possess high positive predictive value (PPV), negative predictive values (NPV), and specificity to diagnose rejection and predict prognosis (Dharnidharka and Malone, 2018). Serum creatinine of more than 3 mg/dL and spot urine protein to creatinine ratio of more than 1 g/g were associated with increased risk of allograft loss (Redfield et al., 2016). However, the elevation of serum creatinine can occur under a variety of circumstances, and the serum creatinine has a low specificity of 30% for rejection (Anglicheau et al., 2016). In one study, 24-h proteinuria of 1.0 g or more had excellent specificity of 85–91% and low sensitivity of 21–32% for intragraft pathology in an indication biopsy (Naesens et al., 2016). The presence of DSA, especially dnDSA, is associated with a high risk of ABMR and graft loss (Mohan et al., 2012; Aubert et al., 2017). The higher the level of DSA at baseline, the higher the incidence of active ABMR and graft loss (Lefaucheur et al., 2010; Schinstock et al., 2017). Monitoring for dnDSA is recommended in the event of reduced immune suppression, medication nonadherence, or rejection (Schinstock et al., 2020). However, single-antigen flow-beads (SAFB) with Luminex testing had a PPV of 31.6%, a sensitivity of 75.0%, and a specificity of 86.2% (Lefaucheur et al., 2010). DSA analysis may predict the risk of developing ABMR, but the diagnostic value for ABMR is likely to be low. In a recent study, 21.4% of grafts with simultaneous dnDSA and ABMR failed during a mean follow-up period of 4.2 years, and no graft failure occurred in grafts with only dnDSA (Schinstock et al., 2017). The incidence of chronic AMBR was not significantly dependent on the MFI value (Schinstock et al., 2017). These findings may suggest that not all DSAs cause tissue damage. Moreover, problems such as bead saturation, shared epitope, and prozone effect can lead to MFI values that differ from DSA levels, although antibody titration methods can more accurately measure antibody strength (Velidedeoglu et al., 2018; Schinstock et al., 2020).

A variety of promising non-invasive biomarkers have been identified that can be used to monitor the immune status of KT patients: the IFN-γ enzyme-linked immunosorbent spot assay evaluating circulating antidonor T cell alloreactivity, gene expression analysis including Allomap (CareDx, Brisbane, CA), kSORT (ImmuCor, Grand Rapids, MI), Trugraf (ViraCor EuroFins, Lee Summit, MO), urine gene signature panels including Quest Laboratories Renal Transplant Monitoring panel, urinary chemokine including CXCL9 and CXCL10, mircroRNA, and donor-derived cell-free DNA (dd-cfDNA) Allosure (CareDx, Brisbane, CA), Prosepera (Natera, San Carlos, CA) (Dharnidharka and Malone, 2018). Most of these biomarkers are useful for diagnosing or predicting acute rejection or TCMR but still require validation in a large, well-controlled cohort. To date, kSORT and dd-cfDNA are commercially available for acute rejection (Dharnidharka and Malone, 2018). In particular, dd-cfDNA was validated in the Diagnosing Acute Rejection in Kidney Transplant Recipients (DART) study which included patients with active ABMR, chronic active ABMR, or TCMR (Bloom et al., 2017). With a cutoff of 1.0%, dd-cfDNA had a 61% PPV and an 84% NPV for discriminating active rejection and a 44% PPV and a 96% NPV for discriminating ABMR, including chronic active ABMR (Bloom et al., 2017). The combined use of dd-cfDNA and DSA testing improved the non-invasive diagnosis of active ABMR, including chronic active ABMR, with an 81% PPV and an 83% NPV for active ABMR at a 1% threshold of dd-cfDNA (Jordan et al., 2018). The elevation of dd-cfDNA was associated with the development of dnDSA and eGFR decline (Stites et al., 2020). Follow-up of dd-cfDNA should be considered in patients with an increased risk of allograft rejection, including reduced immunosuppressants due to infectious diseases and treatment with immune checkpoint inhibitors for cancer (Hurkmans et al., 2019; Abuzeineh et al., 2020). However, increases in dd-cfDNA are not specific for rejection and increase with graft injury by BK virus-associated nephropathy and urinary tract infection (Knight et al., 2019; Kant et al., 2020). Nevertheless, dd-cfDNA is a promising biomarker, but many questions need to be answered regarding its utility. The Kidney Allograft Outcomes AlloSure Registry (KOAR) study is currently ongoing, and this study may provide more clarity on the ability to detect transplant rejection, its impact on graft outcomes, the performance, and clinical use of dd-cfDNA assays (NCT03326076).

### Future Direction

The understanding of chronic ABMR has developed remarkably. However, chronic ABMR is still the leading cause of kidney allograft loss, and there are still no clearly effective therapeutic regimens. Most of the trials for chronic ABMR, including chronic active ABMR, were small, included heterogeneous patient populations, and could not evaluate treatment responses based on DSA type, complement involvement, and pathological findings. Given the complex immune mechanisms of chronic ABMR, combination therapy may be effective in treating chronic ABMR. It may be necessary to simultaneously prevent antibody production, block the antibody produced, and minimize graft damage. However, strong immune blockade most likely will be associated with side effects, increased risk of infectious diseases, malignancy, and high-costs.

One of the most important questions that patients ask is “How long is my transplant going to work”. Recently, a risk prediction score, iBox, for renal allograft failure was developed to answer this question. The prediction model includes eight functional, histological (including chronic active AMR), and immunological prognostic factors (Loupy et al., 2019). The iBox risk prediction score may be helpful in guiding the patient’s treatment according to the risk, but future research is needed (Loupy et al., 2019).

## Conclusion

In conclusion, the definition of chronic active ABMR continues to evolve, and thus its diagnosis continues to evolve. The diagnosis of chronic active ABMR is no longer simply a histologic diagnosis. The diagnosis now incorporates clinical and molecular factors and is likely to include the addition of biomarkers in the future. There is no known effective preventive or therapeutic treatment. That there is not an effective treatment should not be entirely surprising since even the definition of chronic active ABMR is elusive. Our responsibility is to treat what we can and not to treat what we cannot-to do no harm. Or to use a more contemporary expression, “You’ve got to know when to hold ’em. Know when to fold ’em. Know when to walk away. And know when to run (https://en.wikipedia.org/wiki/The_Gambler_(song))
